# Breeding for resistances to *Ralstonia solanacearum*

**DOI:** 10.3389/fpls.2014.00715

**Published:** 2014-12-12

**Authors:** Gaëlle Huet

**Affiliations:** ^1^INRA, Laboratoire des Interactions Plantes-Microorganismes, UMR441, Castanet-TolosanFrance; ^2^CNRS, Laboratoire des Interactions Plantes-Microorganismes, UMR2594, Castanet-TolosanFrance

**Keywords:** *Ralstonia solanacearum*, control, resistance, breeding, effector

## Abstract

*Ralstonia solanacearum* is one of the most devastating bacterial plant pathogens due to its large host range, worldwide geographic distribution and persistence in fields. This soilborne pathogen is the causal agent of bacterial wilt and it can infect major agricultural crops thereby reducing significantly their yield. To favor infection, the bacterium delivers, through the type three secretion system, effectors that manipulate plant immunity. In this review, the relative efficiency of control strategies and existing resistances to *R*. *solanacearum* will be presented. Then, the genetic and molecular insights gained from the study of bacterial wilt in model plants will be described. Finally, I will explore how the knowledge gathered from unraveling avirulence and virulence mechanisms of *R. solanacearum* effectors could help to develop more durable resistances in crop plants toward this destructive pathogen.

## INTRODUCTION

*Ralstonia solanacearum*, the causal agent of bacterial wilt, is one of the most devastating plant pathogenic bacteria (Mansfield et al., 2012) with a large host range encompassing more than 200 plant species which include major agricultural crops such as tomato, potato and banana (Hayward, 1991; Elphinstone, 2005). *R*. *solanacearum* is a soil-borne bacterium that enters plant roots, invades xylem vessels and spreads rapidly to aerial parts of the plant through the vascular system where its high level of multiplication leads to wilting symptoms and, ultimately, plant death (Genin, 2010). In addition to its lethality, the ability of *R*. *solanacearum* to survive in soils for many years and to form latent infections within indigenous weeds contributes to the difficult eradication of the bacterium (Hayward, 1991; Wenneker et al., 1999). The pathogen is found worldwide, primarily in tropical and subtropical regions (Hayward, 1991) but also in Europe and North America where cold-tolerant strains were introduced in the 1990s (Janse et al., 2004; Swanson et al., 2005). The dissemination of *R*. *solanacearum* is a threat to crops and the pathogen is considered a quarantine bacterium.

*Ralstonia solanacearum* strains present an extensive genetic diversity and are divided in four phylotypes corresponding roughly to the strains’ geographic origin: Asia (phylotype I), the Americas (II), Africa (III), and Indonesia (IV). Phylotype II has two subclusters: IIA and IIB (Fegan and Prior, 2005) and only strains belonging to phylotype IIB are responsible for bacterial wilt of potato in cold and temperate regions (Janse et al., 2004). Phylotypes are not related with host preference as strains from all phylotypes are able to cause disease on potato, tomato, pepper, and eggplant (Cellier and Prior, 2010; Lebeau et al., 2011).

Among the virulence determinants of *R*. *solanacearum*, the type three secretion system (TTSS), a molecular syringe whose structural and regulatory elements are encoded by *hrp* (hypersensitive response and pathogenicity) genes, is essential for pathogenicity (Vasse et al., 2000). TTSS injects effector proteins into plant cells to favor the bacterial infection by subverting and exploiting the host signaling pathways (Poueymiro and Genin, 2009). Effectors could promote nutrient leakage but mostly they are predicted to manipulate plant defenses (Goel et al., 2008; Deslandes and Rivas, 2012).

There are two levels in plant immunity (Jones and Dangl, 2006). The first one uses cell surface pattern recognition receptors (PRRs) to detect pathogen-associated molecular patterns (PAMPs) and initiate PAMP-triggered immunity (PTI). The second involves nucleotide-binding leucine-rich repeat (NB-LRR) proteins, encoded by resistance (*R*) genes, which sense pathogen effectors and elicit a potent immune response called effector-triggered immunity (ETI). ETI is faster, longer and stronger than PTI and usually leads to a local cell death, the hypersensitive response (HR), which stops the spread of the pathogen (Jones and Dangl, 2006). Some effectors that enable the pathogen to overcome PTI are recognized by R proteins and the effector is thus termed an avirulence (Avr) protein (Jones and Dangl, 2006). Disease (susceptibility) results when one or both R/Avr partners are absent. Conversely, resistance ensues when R and cognate Avr effector are both present. The activation of plant defenses by an R protein is termed qualitative resistance and provides a complete disease resistant phenotype. An incomplete resistance (tolerance), leading to the reduction rather than the eradication of the disease, is called quantitative (Young, 1996). Quantitative disease resistance (QDR) molecular characterization is still in its infancy. *R* genes (Roux et al., 2010, 2014; Van der Linden et al., 2013) can contribute to QDR as well as components such as kinases or transporters (St Clair, 2010; Roux et al., 2014).

Continuous increase in food production is needed to face the world’s population growth. One way to achieve this goal is by sustainably reducing crop losses to pathogens like* R*. *solanacearum.* This review provides a summary on current control strategies of bacterial wilt and highlights the difficulties of breeding for resistance to* R*. *solanacearum.* In addition, knowledge gained from studies in model plants on resistance to bacterial wilt will be discussed. Finally, the use of *R*. *solanacearum* effectors to decipher the molecular mechanisms of plant immunity and identify new sources of resistance will be explored.

## STRATEGIES FOR CONTROL OF *R. solanacearum*

Integrated strategies for control of *R. solanacearum* are complex because the bacterium is able to infect crops as a soil-borne, water-borne, or seed/tuber-borne organism. Therefore, to avoid the dissemination of *R. solanacearum*, it is recommended to plant healthy seeds in pathogen free soil combined with irrigation water known to be free of *R. solanacearum* (French, 1994). In infested soils, crop rotation (2–5 years), control of weed hosts and survey of water for irrigation can reduce the bacterial load (Lopez and Biosca, 2005). Chemical control, in addition to being potentially harmful to the environment, was not proved to be efficient to eradicate *R. solanacearum* (Saddler, 2005; Denny, 2006). This can be explained by the bacterium localization in the deeper soil layers or sheltered in xylem vessels of infected plants and weeds (Wenneker et al., 1999). In addition, a soil dependent effect has been observed and therefore soil disinfection is not universally applicable (Saddler, 2005). An alternative control strategy was to use biological control agent such as antagonistic bacteria or avirulent mutants of *R. solanacearum* but the promising results obtained under controlled conditions were not confirmed in the field (Saddler, 2005). The most extensively studied avirulent mutants of *R. solanacearum* are *hrp*^-^ mutant strains that no longer possess a functional TTSS (Frey et al., 1994). *hrp*^-^ mutant strains are still able to multiply, and, likely control bacterial wilt through competition with wild type strains for space and nutrients. Assays of protection by *hrp*^-^ mutants have been conducted on tomato and potato plants but, thus far, they did not help to reduce bacterial wilt in fields (Saddler, 2005; Denny, 2006). In the absence of efficient strategies to eradicate *R. solanacearum* from infected soils and water, the use of resistant cultivars appears to be the best disease control strategy.

## BREEDING FOR RESISTANCE TO *R. solanacearum*

Resistance breeding to *R. solanacearum* in solanaceous crops appears to be regional or linked to climatic conditions (Hayward, 1991) and this limited success is due to all the constraints resistant cultivars must outsmart. First, the breeding must combine durable resistance with desirable agronomic traits. Second, resistant cultivars must be able to face the diversity of agro-ecological zones where the bacteria proliferates and the high genetic variability of *R. solanacearum* strains. Third, breeding for highly resistant cultivars must be prioritized to avoid further *R. solanacearum* dissemination due to tolerant plants that shelter virulent bacteria without showing disease symptoms. Finally, the available sources of resistance were found to be polygenic and, despite the identification of QTLs (quantitative trait loci) controlling resistance to bacterial wilt in tomato (Mangin et al., 1999; Wang et al., 2000, 2013; Carmeille et al., 2006), tobacco (Qian et al., 2013) and eggplant (Lebeau et al., 2013), the development of resistant crops is impeded by the difficulty in transferring into cultivars a high number of genes that, in addition, can be linked to undesirable traits (Denny, 2006).

In potato, high level resistance to bacterial wilt has been identified in *Solanum phureja* species (Sequeira and Rowe, 1969), however, the resistance was unstable across locations and its breakdown was triggered by high temperature and decrease in light intensity (French and De Lindo, 1982). In tomato, the polygenic resistance to bacterial wilt in the resistant cultivar Hawaii 7996 (Grimault et al., 1995) was suggested to be strain specific (Wang et al., 2000) and, more recently, it was hypothesized that QTLs in Hawaii 7996 may deploy a phylotype-specific resistance (Carmeille et al., 2006). These results exemplify the difficulty of obtaining a worldwide resistance to *R. solanacearum.*

## GENETIC BASIS OF RESISTANCES IN MODEL PLANTS

To date, studies on resistance to bacterial wilt in model plants were mainly conducted in *Arabidopsis thaliana* and *Medicago truncatula*. As *R. solanacearum* infects a large number of leguminous plants, such as peanut and common bean, a model pathosystem for the study of *R. solanacearum*-legume interactions was developed with *M. truncatula* (Vailleau et al., 2007). A genetic analysis of recombinant inbred lines identify three QTLs involved in disease resistance and fine mapping showed that the major QTL include a cluster of seven putative *R* genes (Vailleau et al., 2007; Ben et al., 2013). Future functional characterization is required to depict the molecular mechanisms underlying *M. truncatula* resistance to bacterial wilt and to appraise their potential application in breeding.

Several* R. solanacearum* strains are able to cause the wilting of *A. thaliana* plants (Deslandes et al., 1998) and insightful knowledge on the molecular basis of resistance to bacterial wilt was uncovered in this pathosystem. In *A. thaliana* accession Col-0, a polygenic mechanism encompassing three QTLs governs resistance to* R. solanacearum* strain 14.25 (Godiard et al., 2003). In these loci, the LRR receptor-like kinase *ERECTA* involved in development of aerial organs was identified thus suggesting a cross-talk between resistance and developmental pathways (Godiard et al., 2003). Other studies established that resistance in *A. thaliana* to *R. solanacearum* could also be monogenic and identified dominant and recessive loci (Deslandes et al., 1998; Ho and Yang, 1999). Further analysis determined that the recessive *RRS1-R* (Resistance to *Ralstonia Solanacearum* 1) gene encodes an atypical R protein harboring a C-terminal WRKY DNA-binding domain (Deslandes et al., 2002) hypothesized to act as a negative transcriptional regulator of plant defenses (Noutoshi et al., 2005). It’s the recognition by RRS1-R of the bacterial effector PopP2 that triggers resistance in *A. thaliana* accession Nd-1 inoculated with *R. solanacearum* GMI1000 strain (Deslandes et al., 2003). RRS1-R physically associates with another R protein, RPS4 (Resistant to *Pseudomonas Syringae* 4), to cooperatively trigger immunity (Narusaka et al., 2009; Williams et al., 2014). The transfer of the *RRS1/RPS4* pair of *R* genes from *A. thaliana* into tomato was able to confer immunity to *R. solanacearum* (Narusaka et al., 2013). This study demonstrates that interfamily transfer of *R* genes can provide a new strategy to develop pathogen-resistant crops (Narusaka et al., 2013). Surprisingly, PopP2 perception by RRS1 can also lead to *A. thaliana* tolerance to bacterial wilt (Van der Linden et al., 2013). In the accession Kil-0, after inoculation with *R. solanacearum* strain BCCF402, the plants showed no wilting symptoms despite high bacterial numbers (Van der Linden et al., 2013). This work is one of few examples describing that tolerance is not always a polygenic trait and that an *R* gene can be common for both *A. thaliana* resistance and tolerance (Roux et al., 2010; Van der Linden et al., 2013). Van der Linden and co-workers pointed out the risk of *R. solanacearum* persistence in the field after deployment of RRS1 in transgenic crops. Thus, despite a promising interfamily transfer of RRS1/RPS4 in crops (Narusaka et al., 2013), the use of RRS1 as a source of resistance to bacterial wilt may not be a relevant choice.

## EFFECTOR ASSISTED IDENTIFICATION OF RESISTANCES TO *R. solanacearum*

Unraveling the molecular functions of effectors is insightful for a mechanistic understanding of the processes underlying plant immune responses (Deslandes and Rivas, 2012). Despite the fact that *R*. *solanacearum* is one of the most destructive phytopathogens, few of its effectors have been functionally characterized and the nature of their plant targets remains largely unknown [for reviews see (Peeters et al., 2013b; Deslandes and Genin, 2014)]. Some *R*. *solanacearum* effectors have been shown to trigger an HR-like response in petunia (Arlat et al., 1994), *A. thaliana* (Solé et al., 2012; Williams et al., 2014), tobacco (Arlat et al., 1994; Poueymiro et al., 2009; Solé et al., 2012) and eggplant (Nahar et al., 2014). Besides, some *R*. *solanacearum* effectors have been found to be under a strong diversifying positive selection, to contribute to the pathogenicity of the bacteria and to dampen plant defense responses (Peeters et al., 2013b; Deslandes and Genin, 2014). Collectively these data demonstrate that further functional characterization of *R*. *solanacearum* effectors will help to identify pivotal components of plant immunity that are manipulated by, or able to perceive, effectors. In order to gather useful knowledge to transfer in breeding programs, future work on characterizing molecular interactions between effectors and plant components should take place in agronomical relevant plants. For example, research could be conducted on tomato and potato, two major crops, whose genome availability should facilitate functional studies.

The availability of genome sequences from eleven *R*. *solanacearum* strains, belonging to the four phylotypes, allowed to establish the core effector repertoire of the bacterium (Peeters et al., 2013a). Core effectors presumably represent ancestral effectors and can be considered as crucial for the interaction between *R*. *solanacearum* and its hosts (Peeters et al., 2013a). “Effectoromics” refers to a new high-throughput approach that uses effectors for probing plant germplasm to detect *R* genes and to improve their deployment in the field (Vleeshouwers et al., 2011; Vleeshouwers and Oliver, 2014). This useful technique could be applied to identify *R*. *solanacearum* effectors with avirulent function and hasten the discovery of their cognate *R* genes. Besides, by performing plant germplasm screening with core effectors, the chances to identify an *R* gene displaying a broad and durable spectrum of resistance should be increased. Furthermore, effector-assisted breeding also allows us to determine the potential of a new *R* gene for durable resistance after deployment in the field by checking its expanded recognition specificity toward various effector alleles found in the pathogen population (Vleeshouwers and Oliver, 2014). As *R*. *solanacearum* strains present an extensive genetic diversity, the efficiency of an effectoromics approach could be increased by focusing on bacterial strains/plant germplasms systems that have co-evolved jointly in a same geographic area.

## PERSPECTIVES

Thus far, resistances to bacterial wilt available in solanaceous crops behave differently under changing environmental conditions. In addition, *R*. *solanacearum* strains exhibit an extensive genetic diversity worldwide. It thus seems like a chimera to look for universal resistances to bacterial wilt. A more realistic approach would be to seek for sources of resistance adapted only to a given ecosystem. Moreover, an ecological approach of bacterial wilt would enable to establish, in natural ecosystems, the pathogen population profile and monitor its changes. This could potentially lead to the identification of new biological control agents, naturally occurring in the soil microflora in fields, which would be more efficient to compete with *R*. *solanacearum* (Saddler, 2005). Also, a precise knowledge of the endemic *R*. *solanacearum* strains encountered in cultivation areas would guide the deployment of the most relevant resistant cultivars.

The identification of new resistance sources could be accelerated by exploiting *R*. *solanacearum* effectors through an effectoromics approach (Vleeshouwers and Oliver, 2014), as well as through the molecular characterization of their virulence and avirulence functions. Once identified, monogenic over polygenic resistances should be prioritized to facilitate their transfer into crops and provide a higher level of resistance more likely to favor the eradication of *R*. *solanacearum.*

After identifying new resistance sources, to increase the chance of success in achieving durable resistance to bacterial wilt, *R*-gene stacking in fields should be applied (Dangl et al., 2013). Moreover, to reduce the bacterial pressure and decrease resistance breakdown, deployment of resistant cultivars should be backed up with an integrated management strategy to decrease the bacteria survival in soil, water and in the rhizosphere of weedy and native non-host plants (Lopez and Biosca, 2005).

In conclusion, only the elaboration of a complex strategy for resistance and control of *R*. *solanacearum* will be able to fight off this multifaceted pathogen.

## Conflict of Interest Statement

The author declares that the research was conducted in the absence of any commercial or financial relationships that could be construed as a potential conflict of interest.

## References

[B1] Arlat M. Van Gijsegem F. Huet J. C. Pernollet J. C. Boucher C. A. (1994). PopA1, a protein which induces a hypersensitivity-like response on specific petunia genotypes, is secreted via the Hrp pathway of *Pseudomonas solanacearum*. *EMBO J.* 13 543–553. 8313899 10.1002/j.1460-2075.1994.tb06292.x PMC394843

[B2] Ben C. Debellé F. Berges H. Bellec A. Jardinaud M. F. Anson P. (2013). MtQRRS1, an R-locus required for *Medicago truncatula* quantitative resistance to *Ralstonia solanacearum*. *New Phytol.* 199 758–772 10.1111/nph.12299 23638965

[B3] Carmeille A. Caranta C. Dintinger J. Prior P. Luisetti J. Besse P. (2006). Identification of QTLs for *Ralstonia solanacearum* race 3-phylotype II resistance in tomato. *Theor. Appl. Genet.* 113 110–121 10.1007/s00122-006-0277-3 16614830

[B4] Cellier G. Prior P. (2010). Deciphering phenotypic diversity of *Ralstonia solanacearum* strains pathogenic to potato. *Phytopathology* 100 1250–1261 10.1094/PHYTO-02-10-0059 20672871

[B5] Dangl J. L. Horvath D. M. Staskawicz B. J. (2013). Pivoting the plant immune system from dissection to deployment. *Science* 341 746–751 10.1126/science.1236011 23950531 PMC3869199

[B6] Denny T. P. (2006). “Plant pathogenic *Ralstonia* species,” in *Plant-Associated Bacteria* ed. Gnanamanickam S. S. (Dordrecht: Springer) 573–644.

[B7] Deslandes L. Genin S. (2014). Opening the *Ralstonia solanacearum* type III effector tool box: insights into host cell subversion mechanisms. *Curr. Opin. Plant Biol.* 20C, 110–117 10.1016/j.pbi.2014.05.002 24880553

[B8] Deslandes L. Olivier J. Peeters N. Feng D. X. Khounlotham M. Boucher C. (2003). Physical interaction between RRS1-R, a protein conferring resistance to bacterial wilt, and PopP2, a type III effector targeted to the plant nucleus. *Proc. Natl. Acad. Sci. U.S.A.* 100 8024–8029 10.1073/pnas.1230660100 12788974 PMC164706

[B9] Deslandes L. Olivier J. Theulieres F. Hirsch J. Feng D. X. Bittner-Eddy P. (2002). Resistance to *Ralstonia solanacearum* in *Arabidopsis thaliana* is conferred by the recessive rrs1-r gene, a member of a novel family of resistance genes. *Proc. Natl. Acad. Sci. U.S.A.* 99 2404–2409 10.1073/pnas.032485099 11842188 PMC122377

[B10] Deslandes L. Pileur F. Liaubet L. Camut S. Can C. Williams K. (1998). Genetic characterization of rrs1, a recessive locus in *Arabidopsis thaliana* that confers resistance to the bacterial soilborne pathogen *Ralstonia solanacearum*. *Mol. Plant Microbe Interact.* 11 659–667 10.1094/MPMI.1998.11.7.659 9650298

[B11] Deslandes L. Rivas S. (2012). Catch me if you can: bacterial effectors and plant targets. *Trends Plant Sci.* 17 644–655 10.1016/j.tplants.2012.06.011 22796464

[B12] Elphinstone J. G. (2005). “The current bacterial wilt situation: a global view,” in *Bacterial Wilt Disease and the Ralstonia solanacearum Species Complex* eds Allen C. Prior P. Hayward A. C. (Saint Paul, MN: APS Press) 9–28.

[B13] Fegan M. Prior P. (2005). “How complex is the *Ralstonia solanacearum* species complex?” in *Bacterial Wilt Disease and the Ralstonia solanacearum Species Complex* eds Allen C. Prior P. Hayward A. C. (Saint Paul, MN: APS Press) 449–461.

[B14] French E. R. (1994). “Strategies for integrated control of bacterial wilt of potatoes,” in *Bacterial Wilt: The Disease and its Causative Agent, Pseudomonas solanacearum* eds Hayward A. C. Hartman G. L. (Wallingford: CAB International) 199–207.

[B15] French E. R. De Lindo L. (1982). Resistance to *Pseudomonas solanacearum* in potato: specificity and temperature sensitivity. *Phytopathology* 72 1408–1412 10.1094/Phyto-72-1408

[B16] Frey P. Prior P. Marie C. Kotoujansky A. Trigalet-Demery D. Trigalet A. (1994). Hrp^-^ mutants of *Pseudomonas solanacearum* as potential biocontrol agents of tomato bacterial wilt. *Appl. Environ. Microbiol.* 60 3175–3181. 16349373 10.1128/aem.60.9.3175-3181.1994 PMC201786

[B17] Genin S. (2010). Molecular traits controlling host range and adaptation to plants in *Ralstonia solanacearum*. *New Phytol.* 187 920–928 10.1111/j.1469-8137.2010.03397.x 20673287

[B18] Godiard L. Sauviac L. Torii K. U. Grenon O. Mangin B. Grimsley N. H. (2003). Erecta, an LRR receptor-like kinase protein controlling development pleiotropically affects resistance to bacterial wilt. *Plant J.* 36 353–365 10.1046/j.1365-313X.2003.01877.x 14617092

[B19] Goel A. K. Lundberg D. Torres M. A. Matthews R. Akimoto-Tomiyama C. Farmer L. (2008). The *Pseudomonas syringae* type III effector HopAM1 enhances virulence on water-stressed plants. *Mol. Plant Microbe Interact.* 21 361–370 10.1094/MPMI-21-3-0361 18257685

[B20] Grimault V. Prior P. Anais G. (1995). A monogenic dominant resistance of tomato to bacterial wilt in hawaii 7996 is associated with plant colonization by *Pseudomonas solanacearum*. *J. Phytopathol.* 143 349–352 10.1111/j.1439-0434.1995.tb00274.x

[B21] Hayward A. C. (1991). Biology and epidemiology of bacterial wilt caused by *Pseudomonas solanacearum*. *Annu. Rev. Phytopathol.* 29 65–87 10.1146/annurev.py.29.090191.000433 18479193

[B22] Ho G. D. Yang C. H. (1999). A single locus leads to resistance of *Arabidopsis thaliana* to bacterial wilt caused by *Ralstonia solanacearum* through a hypersensitive-like response. *Phytopathology* 89 673–678 10.1094/PHYTO.1999.89.8.673 18944680

[B23] Janse J. Beld D. van Den H. (2004). Introduction to europe of *Ralstonia solanacearum* biovar 2, race 3 in *Pelargonium zonale* cuttings. *J. Plant Pathol.* 86 147–155.

[B24] Jones J. D. Dangl J. L. (2006). The plant immune system. *Nature* 444 323–329 10.1038/nature05286 17108957

[B25] Lebeau A. Daunay M. C. Frary A. Palloix A. Wang J. F. Dintinger J. (2011). Bacterial wilt resistance in tomato, pepper, and eggplant: genetic resources respond to diverse strains in the *Ralstonia solanacearum* species complex. *Phytopathology* 101 154–165 10.1094/PHYTO-02-10-0048 20795852

[B26] Lebeau A. Gouy M. Daunay M. C. Wicker E. Chiroleu F. Prior P. (2013). Genetic mapping of a major dominant gene for resistance to *Ralstonia solanacearum* in eggplant. *Theor. Appl. Genet.* 126 143–158 10.1007/s00122-012-1969-5 22930132

[B27] Lopez M. M. Biosca E. G. (2005). “Potato bacterial wilt management: new prospects for an old problem,” in *Bacterial Wilt Disease and the Ralstonia solanacearum Species Complex* eds Allen C. Prior P. Hayward A. C. (Saint Paul, MN: APS Press) 205–224.

[B28] Mangin B. Thoquet P. Olivier J. Grimsley N. H. (1999). Temporal and multiple quantitative trait loci analyses of resistance to bacterial wilt in tomato permit the resolution of linked loci. *Genetics* 151 1165–1172. 10049932 10.1093/genetics/151.3.1165 PMC1460533

[B29] Mansfield J. Genin S. Magori S. Citovsky V. Sriariyanum M. Ronald P. (2012). Top 10 plant pathogenic bacteria in molecular plant pathology. *Mol. Plant Pathol.* 13 614–629 10.1111/j.1364-3703.2012.00804.x 22672649 PMC6638704

[B30] Nahar K. Matsumoto I. Taguchi F. Inagaki Y. Yamamoto M. Toyoda K. (2014). *Ralstonia solanacearum* type III secretion system effector Rip36 induces a hypersensitive response in the nonhost wild eggplant *Solanum torvum*. *Mol. Plant Pathol.* 15 297–303 10.1111/mpp.12079 24745046 PMC6638889

[B31] Narusaka M. Kubo Y. Hatakeyama K. Imamura J. Ezura H. Nanasato Y. (2013). Interfamily transfer of dual NB-LRR genes confers resistance to multiple pathogens. *PLoS ONE* 8:e55954 10.1371/journal.pone.0055954 PMC3577827 23437080

[B32] Narusaka M. Shirasu K. Noutoshi Y. Kubo Y. Shiraishi T. Iwabuchi M. (2009). Rrs1 and rps4 provide a dual resistance-gene system against fungal and bacterial pathogens. *Plant J.* 60 218–226 10.1111/j.1365-313X.2009.03949.x 19519800

[B33] Noutoshi Y. Ito T. Seki M. Nakashita H. Yoshida S. Marco Y. (2005). A single amino acid insertion in the wrky domain of the *Arabidopsis* TIR-NBS-LRR-WRKY-type disease resistance protein SLH1 (sensitive to low humidity 1) causes activation of defense responses and hypersensitive cell death. *Plant J.* 43 873–888 10.1111/j.1365-313X.2005.02500.x 16146526

[B34] Peeters N. Carrère S. Anisimova M. Plener L. Cazalé A. C. Genin S. (2013a). Repertoire, unified nomenclature and evolution of the type III effector gene set in the *Ralstonia solanacearum* species complex. *BMC Genomics* 14:859 10.1186/1471-2164-14-859 PMC3878972 24314259

[B35] Peeters N. Guidot A. Vailleau F. Valls M. (2013b). *Ralstonia solanacearum*, a widespread bacterial plant pathogen in the post-genomic era. *Mol. Plant Pathol.* 14 651–662 10.1111/mpp.12038 23718203 PMC6638647

[B36] Poueymiro M. Cunnac S. Barberis P. Deslandes L. Peeters N. Cazale-Noel A. C. (2009). Two type III secretion system effectors from *Ralstonia solanacearum* GMI1000 determine host-range specificity on tobacco. *Mol. Plant Microbe Interact.* 22 538–550 10.1094/MPMI-22-5-0538 19348572

[B37] Poueymiro M. Genin S. (2009). Secreted proteins from *Ralstonia solanacearum*: a hundred tricks to kill a plant. *Curr. Opin. Microbiol.* 12 44–52 10.1016/j.mib.2008.11.008 19144559

[B38] Qian Y. L. Wang X. S. Wang D. Z. Zhang L. N. Zu C. L. Gao Z. L. (2013). The detection of QTLs controlling bacterial wilt resistance in tobacco (*N. tabacum* l.). *Euphytica* 192 259–266 10.1007/s10681-012-0846-2

[B39] Roux F. Gao L. Bergelson J. (2010). Impact of initial pathogen density on resistance and tolerance in a polymorphic disease resistance gene system in *Arabidopsis thaliana*. *Genetics* 185 283–291 10.1534/genetics.109.112383 20142437 PMC2870963

[B40] Roux F. Voisin D. Badet T. Balagué C. Barlet X. Huard-Chauveau C. (2014). Resistance to phytopathogens *e tutti quanti*: placing plant quantitative disease resistance on the map. *Mol. Plant Pathol.* 15 427–432 10.1111/mpp.12138 24796392 PMC6638617

[B41] Saddler G. S. (2005). “Management of bacterial wilt disease,” in *Bacterial Wilt Disease and the Ralstonia solanacearum Species Complex* eds Allen C. Prior P. Hayward A. C. (Saint Paul, MN: APS press) 121–132.

[B42] Sequeira L. Rowe P. R. (1969). Selection and utilization of *Solanum phureja* clones with high resistance to different strains of *Pseudomonas solanacearum*. *Am. Potato J.* 46 451–462 10.1007/BF02862028

[B43] Solé M. Popa C. Mith O. Sohn K. H. Jones J. D. Deslandes L. (2012). The awr gene family encodes a novel class of *Ralstonia solanacearum* type III effectors displaying virulence and avirulence activities. *Mol. Plant Microbe Interact.* 25 941–953 10.1094/MPMI-12-11-0321 22414437

[B44] St Clair D. A. (2010). Quantitative disease resistance and quantitative resistance loci in breeding. *Annu. Rev. Phytopathol.* 48 247–268 10.1146/annurev-phyto-080508-081904 19400646

[B45] Swanson J. K. Yao J. Tans-Kersten J. Allen C. (2005). Behavior of *Ralstonia solanacearum* race 3 biovar 2 during latent and active infection of geranium. *Phytopathology* 95 136–143 10.1094/PHYTO-95-0136 18943982

[B46] Vailleau F. Sartorel E. Jardinaud M. F. Chardon F. Genin S. Huguet T. (2007). Characterization of the interaction between the bacterial wilt pathogen *Ralstonia solanacearum* and the model legume plant *Medicago truncatula*. *Mol. Plant Microbe Interact.* 20 159–167 10.1094/MPMI-20-2-0159 17313167

[B47] Van der Linden L. Bredenkamp J. Naidoo S. Fouché-Weich J. Denby K. J. Genin S. (2013). Gene-for-gene tolerance to bacterial wilt in *Arabidopsis*. *Mol. Plant Microbe Interact.* 26 398–406 10.1094/MPMI-07-12-0188-R 23234403

[B48] Vasse J. Genin S. Frey P. Boucher C. Brito B. (2000). The hrpB and hrpG regulatory genes of *Ralstonia solanacearum* are required for different stages of the tomato root infection process. *Mol. Plant Microbe Interact.* 13 259–267 10.1094/MPMI.2000.13.3.259 10707351

[B49] Vleeshouwers V. G. Oliver R. P. (2014). Effectors as tools in disease resistance breeding against biotrophic, hemibiotrophic, and necrotrophic plant pathogens. *Mol. Plant Microbe Interact.* 27 196–206 10.1094/MPMI-10-13-0313-IA 24405032

[B50] Vleeshouwers V. G. Raffaele S. Vossen J. H. Champouret N. Oliva R. Segretin M. E. (2011). Understanding and exploiting late blight resistance in the age of effectors. *Annu. Rev. Phytopathol.* 49 507–531 10.1146/annurev-phyto-072910-095326 21663437

[B51] Wang J. F. Ho F. I. Truong H. T. H. Huang S. M. Balatero C. H. Dittapongpitch V. (2013). Identification of major QTLs asociated with stable resistance of tomato cultivar “Hawaii 7996” to *Ralstonia so*lanacearum. *Euphytica* 190 241–252 10.1007/s10681-012-0830-x

[B52] Wang J. F. Olivier J. Thoquet P. Mangin B. Sauviac L. Grimsley N. H. (2000). Resistance of tomato line hawaii7996 to *Ralstonia solanacearum* Pss4 in Taiwan is controlled mainly by a major strain-specific locus. *Mol. Plant Microbe Interact.* 13 6–13 10.1094/MPMI.2000.13.1.6 10656580

[B53] Wenneker M. Verdel M. Groeneveld R. Kempenaar C. van Beuningen A. Janse J. (1999). *Ralstonia* (*Pseudomonas*) *solanacearum* race 3 (biovar 2) in surface water and natural weed hosts: first report on stinging nettle (*Urtica dioica*). *Eur. J. Plant pathol.* 105 307–315 10.1023/A:1008795417575

[B54] Williams S. J. Sohn K. H. Wan L. Bernoux M. Sarris P. F. Segonzac C. (2014). Structural basis for assembly and function of a heterodimeric plant immune receptor. *Science* 344 299–303 10.1126/science.1247357 24744375

[B55] Young N. D. (1996). QTL mapping and quantitative disease resistance in plants. *Annu. Rev. Phytopathol.* 34 479–501 10.1146/annurev.phyto.34.1.479 15012553

